# A Multimodal AI Framework for Medical Education: Integrating Adaptive Image Retrieval, Fast Synthesis, and LLM-Based Clinical Auditing

**DOI:** 10.3390/jimaging12090438

**Published:** 2026-09-11

**Authors:** Miguel Díaz-Benito, Cecilia Diana-Albelda, Álvaro García-Martín, Mario Rubén Paz Campos, Jesus Bescos

**Affiliations:** 1 Department of Signal Theory and Communications, Universidad Carlos III de Madrid, 28911 Leganés, Spain; 2 Video Processing and Understanding Lab, Escuela Politécnica Superior, Universidad Autónoma de Madrid, 28049 Madrid, Spain; cecilia.diana@uam.es (C.D.-A.); j.bescos@uam.es (J.B.); 3 Hospital Universitario de La Princesa, 28006 Madrid, Spain; juniorpm17@gmail.com

**Keywords:** medical education, image retrieval, diffusion models, multimodal language models, Auto-α, LCM-LoRA, clinical audit

## Abstract

Access to reliable medical images is essential for clinical training. To address this need, this paper presents an extended version of MIRAGE, a multimodal retrieval and generation system that utilizes a shared latent space to process medical queries by retrieving real images from the ROCO dataset, generating synthetic scans, and providing LLM-based clinical descriptions alongside dual-concept visual comparisons. To overcome previous computational limits and the lack of clinical validation, we introduce three core enhancements: first, an Auto-α module to dynamically weight visual and textual similarities; second, the integration of LCM-LoRA to accelerate synthetic image generation; and third, an automated clinical auditor based on Gemini 2.5 Flash. Experimental results demonstrate that Auto-α improves retrieval accuracy for heterogeneous queries, reaching 38.83% Top-1 Recall over a 65,419-image gallery and outperforming nine fusion baselines evaluated under a unified configuration, with a controlled ablation attributing most of this gain to learning the weight rather than merely making it query-adaptive, while the LCM-LoRA module reduces computational costs by a factor of 12.5× in CPU environments, with a blinded radiologist evaluation confirming only a small drop in clinical quality. Furthermore, the clinical auditor achieves a 0.805 Pearson correlation against an expert radiologist, effectively correcting the systematic overestimation of traditional CLIP scores. Finally, the optimized platform is publicly deployed on Hugging Face.

## 1. Introduction

Thanks to the rapid integration of artificial intelligence into healthcare, research has opened the door to the development of accessible and reliable educational tools for medical students. While traditional resources like anatomical atlases are static, modern internet search engines frequently yield unverified or decontextualized images [1].

To address these limitations, multimodal learning has emerged as a powerful approach in healthcare [2,3]. Frameworks mapping visual and textual information into shared latent spaces have significantly improved zero-shot medical image retrieval [4,5,6], as demonstrated by models like MedCLIP [7], which adapts the CLIP architecture [8] for the medical domain. Furthermore, integrating LLMs such as BioGPT [9] and Dolly-v2 [10] allows for the generation of enriched, context-aware clinical descriptions. Alongside retrieval, synthetic image generation via diffusion models [11] provides a practical and ethical solution to data scarcity caused by privacy constraints, enabling the visualization of rare conditions and tailored learning representations [12].

Despite these technological advances, most AI-driven tools applied to medical education and self-directed learning remain text-centric [13,14,15,16,17,18]. There is a significant lack of accessible platforms that seamlessly unify image-based training with generative text.

To bridge this gap, we initially introduced MIRAGE [19], an educational framework built upon the ROCO (Radiology Objects in COntext) dataset [20]. This baseline system integrated semantic medical image retrieval, enriched textual descriptions, and synthetic image generation from user prompts. It also featured a dual-query functionality utilizing latent space arithmetic to allow students to contrast different clinical concepts.

This article is an extended version of the aforementioned work [19]. While the preliminary system demonstrated the potential of multimodal AI in medical education, this paper expands the framework to address its previous computational limitations and lack of clinical validation. The aim that guides this extension, together with the specific objectives and the contributions through which we pursue each of them, is set out in Section 2.

## 2. Aims and Objectives

The aim of this work is to give medical students a single, freely accessible tool that answers a visual question about radiology from end to end. To do so, it finds a real and verified image, explains it in context, generates a synthetic representation, and tells the student how far the result can be trusted. The emphasis is on the system as a whole rather than on any component in isolation. This shapes two decisions that run through the paper: the platform must remain usable on free, CPU-powered infrastructure, and it must cover the whole body rather than one organ or one imaging device, because students move between topics and express the same clinical concept in very different ways. To reach that aim, this extended version of MIRAGE [19] pursues four objectives, each addressed by one of the contributions of this article:

- O1. Make retrieval robust to how the query is written. Rather than asking the user to weight visual and textual evidence, the system should infer that balance for each query. We therefore transition from a fixed embedding-based similarity to a dynamic multimodal retrieval mechanism: while we still allow users to manually balance textual and visual modalities, we introduce Auto-α, a confidence-aware module that automatically and dynamically weights visual and textual similarities on a case-by-case basis. We assess it by comparing Auto-α against nine baselines under a single unified configuration, and by isolating the contribution of each component through a controlled ablation.
- O2. Bring synthetic image generation within reach of CPU-only hardware. Diffusion pipelines are the main computational bottleneck of the system. To overcome the high computational costs associated with them in CPU-powered environments, we integrate an LCM-LoRA adapter into the generative model, drastically reducing the required inference steps and latency. We measure latency and computational cost before and after this integration, and verify that the acceleration does not compromise clinical value through a blinded evaluation by an expert radiologist.
- O3. Tell the student when a retrieved image does not match the query. We incorporate an automated evaluation module powered by state-of-the-art multimodal LLMs to act as a clinical “second opinion,” assessing the alignment of retrieved query-image pairs. To guarantee clinical accuracy, its outputs are validated against the annotations of a radiologist, measuring correlation, absolute agreement and systematic bias.
- O4. Keep the whole system deployable and open. The framework is released as a ready-to-use public interface requiring no installation. We broaden its accessibility and interactivity through comprehensive educational features, including a virtual teacher chatbot, interactive quizzes, and automated multilingual support, so that it functions as a learning environment.

## 3. Related Work

### 3.1. Adaptive Multimodal Retrieval

MIRAGE [19] was built on the ROCO dataset and used a medical fine-tuning of CLIP [21] to embed images and captions into a single latent space. A student query was encoded with the same model and matched against the precomputed image embeddings by cosine similarity; the captions of the top matches were then passed to Dolly-v2-3b [10], which produced an enriched description of the concept, and that description was re-encoded and used for a second retrieval pass. In parallel, the original prompt was fed to Prompt2MedImage [22], a latent diffusion model trained on medical content, to synthesise an image that the student could compare against the real one. A dual-search mode applied arithmetic directly in the latent space, subtracting the embedding of one concept and adding another, so that two related conditions could be retrieved and displayed side by side. The whole pipeline ran on the free GPU tier of Kaggle without any local installation, answering a query in roughly 40 to 60 s, and was validated by measuring cosine similarity between matched and mismatched text and image pairs, reaching classification accuracies between 97% and 99%.

That first version left three problems open, and they motivate the first three objectives stated above. Retrieval relied on a single similarity computed in one direction, with no mechanism to balance visual against textual evidence depending on how the query was phrased, and the indexed gallery covered only about 5% of ROCO because of memory constraints. Generation depended on a standard diffusion pipeline that required a GPU, which limited where the platform could be hosted. And the evaluation, although quantitative, rested entirely on embedding-space similarity and qualitative inspection: no clinician had assessed whether the retrieved or generated images were clinically appropriate, and the system had no way of warning a student when a retrieved image did not match the query.

Other studies present cross-modality retrieval approaches [23,24,25,26]. Especially in healthcare, which often involves technical terminology and complex concepts, adaptive strategies that take advantage of all available data modalities have become essential [2,27].

Alternatively, recent works propose per-query adaptive fusion [28,29,30,31], where weights are derived from similarity score distributions, entropy, or other different concepts. For example, several methods estimate feature or modality reliability from the tail shape of the similarity curve or its area, and adapt fusion weights [32,33]. Entropy schemes dynamically reweight features via PageRank-style optimization, yielding consistent gains over fixed-weight baselines [34,35]. Related approaches in cross-modality and multimodal settings perform query-aware late fusion of modality-specific similarities, improving robustness under heterogeneous queries and noisy modalities [36,37], just as multi-scale feature fusion enhances noise resistance in medical image retrieval [38].

### 3.2. Efficient Synthetic Image Generation

Image generation techniques using Deep Learning approaches are continuously improving, offering advantages across various disciplines. In the medical field, where gathering real datasets is hindered by privacy and consent concerns, synthetic imagery is a practical workaround [12], especially when dealing with rare diseases. Additionally, it facilitates the design of personalized visual aids for medical education, as these visual examples are essential for deep comprehension and learning [39,40].

However, the generation of images using standard diffusion models is computationally expensive [41,42,43]. Therefore, recent literature has explored new techniques to accelerate this process. In this domain, parameter-efficient approaches such as Low-Rank Adaptation (LoRA) [44] and Latent Consistency Models (LCM) [45,46], or a combination of both [46,47], aim to reduce the number of inference steps required to create these synthetic images.

While these parameter-efficient and consistency-based models address the computational cost, their implementation can negatively impact the visual fidelity of generated images [48]. Reducing inference steps frequently compromises overall quality, potentially omitting details required in medical imagery [48]. Future directions [49] include improving sampling efficiency and model compression in diffusion-based image restoration.

### 3.3. Automated Evaluation and Clinical Validation

Historically, the evaluation of retrieved and AI-generated images relied heavily on pixel-level similarities or lexical overlap metrics. While these traditional approaches provided quantitative baselines, they failed to capture clinical semantics and anatomical correctness [50,51]. To address this limitation, the field shifted towards representation learning [7]. The development of metrics like the CLIP score, which evaluates the semantic alignment between two elements within a shared latent space, has significantly improved the assessment of both image retrieval and synthetic generation [52,53].

However, the use of metrics like the CLIP score to assess quality often lacks true clinical validation [54]. Furthermore, standard automated metrics fail to account for the ambiguity and poor quality of certain medical images, which have been shown to systematically confound both AI models and human experts [55]. Consequently, recent literature has increasingly adopted an “LLM-as-a-judge” paradigm [56,57,58], where state-of-the-art multimodal models [59,60] are being deployed as automated tools to score the clinical relevance of medical outputs [57,61].

## 4. Materials and Methods

### 4.1. System Overview

**Figure 1:**
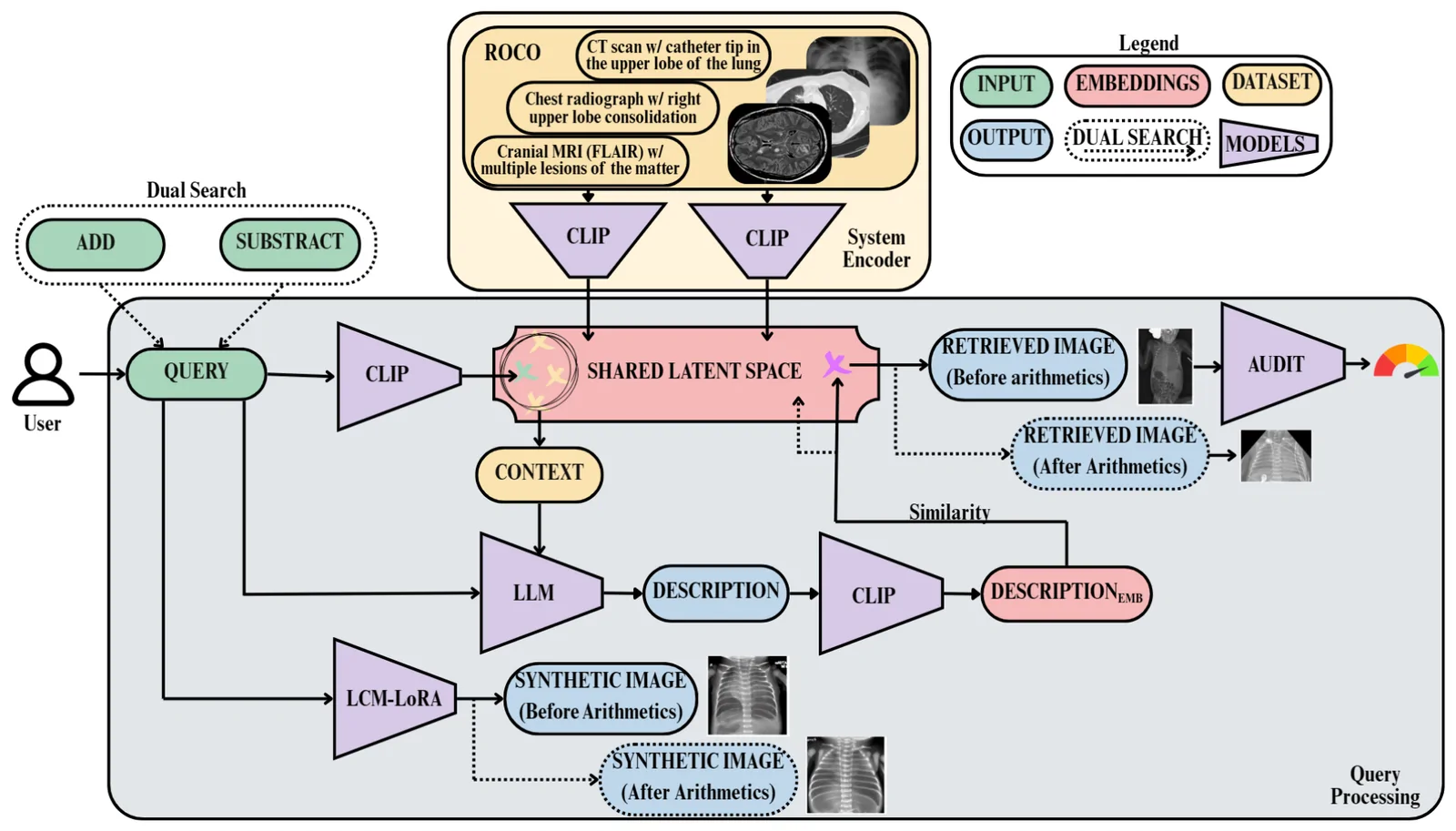
General system pipeline. The input text query is embedded into a shared semantic space and matched with the ROCO dataset to retrieve and audit the most relevant actual image, generate a comprehensive text description, and synthesize a new artificial medical scan using LCM-LoRA. The framework also enables side-by-side clinical comparisons through latent space mathematics.

To overcome the limitations of zero-shot medical image retrieval systems like MIRAGE [19], we present a new multimodal framework, illustrated in Figure 1. In this workflow, a user query is transformed into a latent space representation using a CLIP model fine-tuned on the ROCO dataset. The most similar embeddings based on cosine similarity are retrieved; their corresponding captions, along with the initial query of the user, are then fed into an LLM to generate an enriched description of the concept. This description is subsequently mapped back into the embedding space to retrieve the most relevant concept in the database. In order to ensure the quality of the retrieved image, we developed an auditing system where a multimodal LLM receives the retrieved image and the query of the user and evaluates how accurate the retrieval was. Additionally, the user query is utilized to generate a synthetic image representing the clinical concept. Finally, the framework supports dual-search functionality, allowing concepts to be added and subtracted within the latent space to facilitate a direct comparison between different retrieved items. This system is deployed on Hugging Face, an open-access platform, making it freely available to the community through a ready-to-use interface.

The system is built upon the Radiology Objects in Context (ROCO) dataset, a multimodal medical repository extracted from the Open Access subset of PubMed Central. Since the data come primarily from peer-reviewed scientific journals, they offer a reliable baseline of academic rigor and medical context. In this work, we utilized the training set as our data source, which consists of more than 65,000 image-caption pairs encompassing diverse medical modalities, including computed tomography (CT), X-ray, magnetic resonance imaging (MRI), and ultrasound, among others. Because the collection is assembled from the open-access biomedical literature, these pairs originate from thousands of independent publications and therefore from a large number of institutions, scanners and acquisition protocols worldwide, rather than from a single hospital or a single device, and they span the whole body rather than one anatomical region. We mapped these images and captions into a shared latent space using an open-access, fine-tuned vision–language model [21].

The workflow begins when a user submits a text query describing a medical concept they wish to study. First, the system processes this text using the CLIP model to convert it into a latent representation. Then, a retrieval process searches within the latent space to find the most similar concept. In this work, we propose and evaluate different retrieval approaches, allowing the user to choose their preferred method. Through this retrieval system, the user receives the real medical image that best matches their input query.

An important new feature added to this workflow is the clinical audit of the retrieved image from the real database. The goal is to provide the user with a “second opinion” to assess the quality and relevance of the retrieved image compared to their original query. To perform this audit, we use a multimodal LLM, where, by analyzing both the text query of the user and the retrieved image, the LLM rates the quality of the match on a scale from 0 to 100 and provides a brief justification for its score.

**Figure 2:**
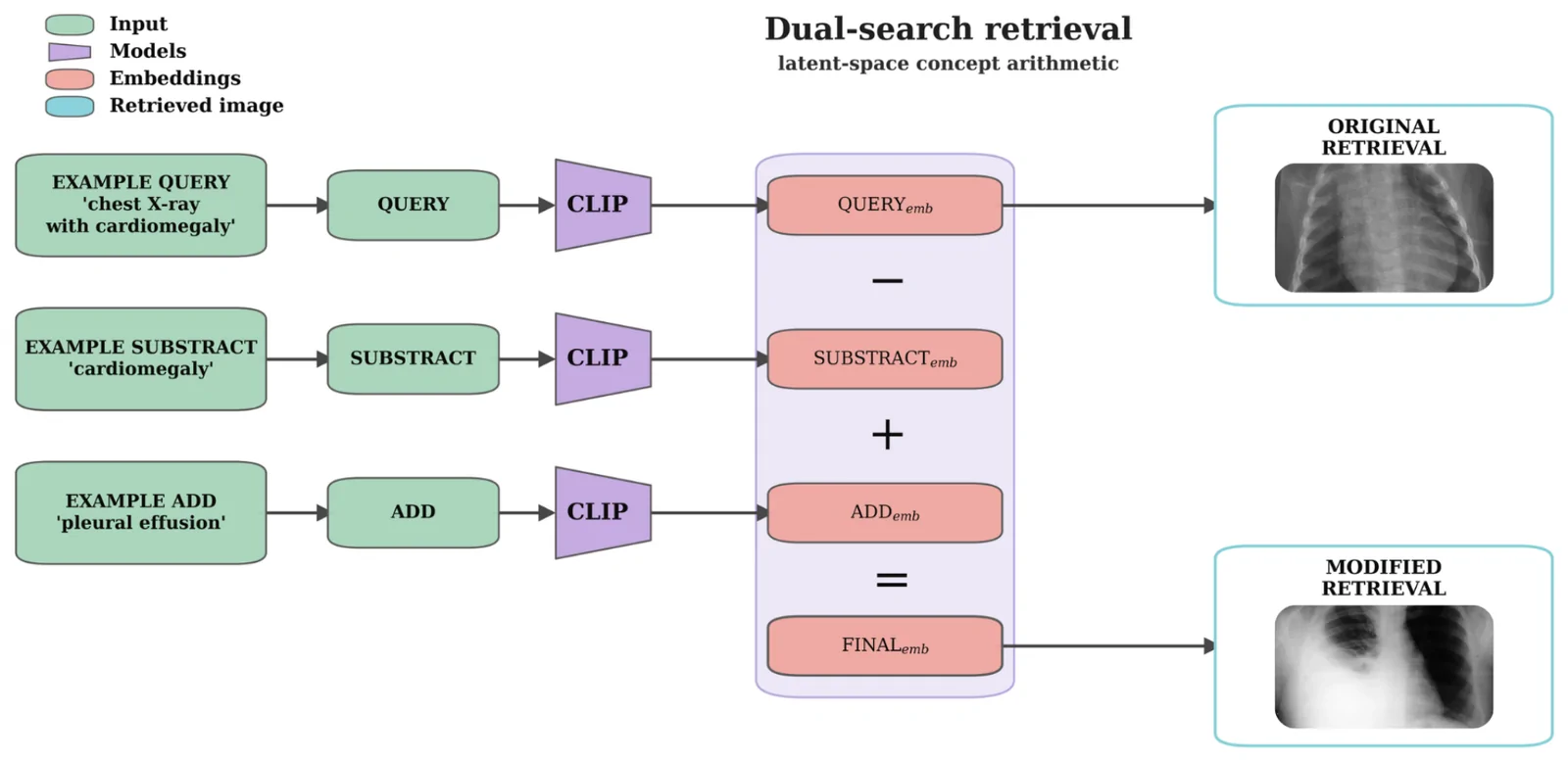
Workflow of the dual search functionality. By applying latent space arithmetic to add or subtract specific clinical concepts, the system retrieves modified medical images, facilitating a side-by-side visual comparison of different pathologies or anatomical features.

Another relevant feature of the system is the “dual search,” which allows users to input a baseline query and specify concepts they want to change. As Figure 2 illustrates, users can choose to add or subtract specific medical terms. The system performs this addition and subtraction mathematically, directly within the latent space. For instance, a student could start with a query for a “chest X-ray with cardiomegaly”, subtract “cardiomegaly”, and add “pleural effusion” to contrast the visual differences between heart enlargement and fluid accumulation. Afterward, the user can simultaneously view and compare the images retrieved before and after the modification, as illustrated in Figure 2.

In order to provide relevant context about the concept that the user wants to explore, once the system retrieves the most similar image-caption pairs, it does not display the captions as isolated text. Instead, the framework uses them as contextual input for an LLM that generates a comprehensive description of the queried concept. This step helps the user achieve an overall understanding of the topic.

Finally, because visual examples are essential for learning [39], we developed a module to generate synthetic medical images directly from user prompts. This feature aims to be a practical alternative to real data, especially when the concept is rare or underrepresented in the database. To produce these images, the framework feeds the query of the user into Prompt2MedImage [22], an open-access latent diffusion-based model explicitly obtained by fine-tuning Stable Diffusion on ROCO. By presenting both a real retrieved image and a synthetically generated one, the system allows students to compare visual features side-by-side.

However, the use of diffusion models is computationally demanding, especially when the platform is CPU-powered. Therefore, to make this process faster and ease the use of the platform, we optimized the pipeline by incorporating an LCM-LoRA (Latent Consistency Model-Low-Rank Adaptation) [47] adapter. This optimization accelerates the diffusion process by reducing the number of inference steps required to create the image.

### 4.2. Retrieval Optimization

#### 4.2.1. Cross-Modality Retrieval

Classical retrieval systems rely on straightforward approaches to retrieve the image that best matches the queried text. When a user inputs a query, the system first converts it into a latent space embedding ($e_{q}$). This query embedding is then compared against the collection of image embeddings ($E_{img}$) stored in our database. To identify the most relevant result, we measure the match between the query and all available images using a standard cosine similarity score, as expressed in Equation (1). Since all query and gallery embeddings are L2-normalized, this inner product is equivalent to the cosine similarity.

While this traditional approach works well in general domains, medical queries often contain complex and specific terminology, making it challenging to retrieve an image based only on the visual embeddings of the database. To bridge this modality gap between text and images, we introduce a hybrid fusion strategy. Instead of relying just on visual information, this approach also compares the query of the user against the original textual captions associated with the images in the dataset (see Equation (1)).

(1) $$S_{visual}=e_{q}\cdot E_{img}^{T},\;S_{txt}=e_{q}\cdot E_{txt}^{T}$$

By using both modalities (the images and their corresponding captions) we can evaluate how similar the query of the user is to both the visual and textual information in the database. To integrate these two similarity scores, we propose two different approaches.

#### 4.2.2. Approach 1: User-Tunable Weighted Fusion

Our first solution gives control back to the user through a simple tuning parameter, α, which ranges from 0 to 1. The final retrieval score is a tuned combination of the visual score and the text score, following the formulation in Equation (2).

(2) $$S_{fused}=\alpha \;S_{visual}+\left(1-\alpha \right)\;S_{txt}$$

**Figure 3:**
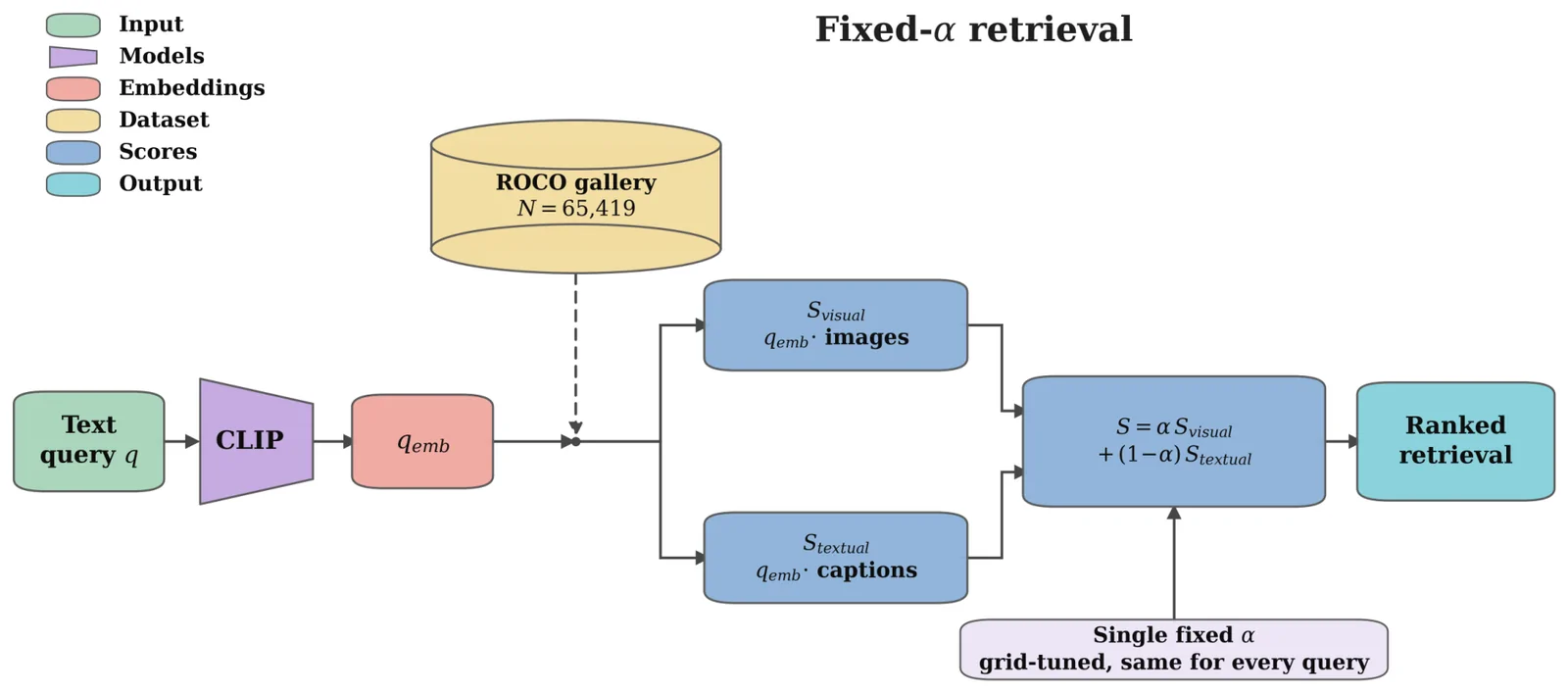
Pipeline of the cross-modality retrieval, where each modality is weighted with a tunable hyperparameter based on the preferences of the user.

With this approach we aim to increase the flexibility of the system, giving the user complete control of the source of information to obtain the retrieved image. For example, if a user sets α=0.0, the system ignores the visual features and searches only by text. On the other hand, choosing an intermediate value (like α=0.5) may help when both the clinical description and the visual pattern are equally important to retrieve the right case. The pipeline used for this approach is illustrated in Figure 3.

#### 4.2.3. Approach 2: Auto-α (Confidence-Aware Fusion)

Although providing a hyperparameter to tune the retrieval allows for user customization, requiring users to estimate the optimal weight for each search is often impractical. To address this, we developed an automated alternative. While adaptive fusion mechanisms have been explored in other domains, their application remains largely unexplored in zero-shot medical retrieval [34,62,63]. Therefore, we propose Auto-α, a lightweight module that predicts the optimal fusion weight for each specific query.

**Figure 4:**
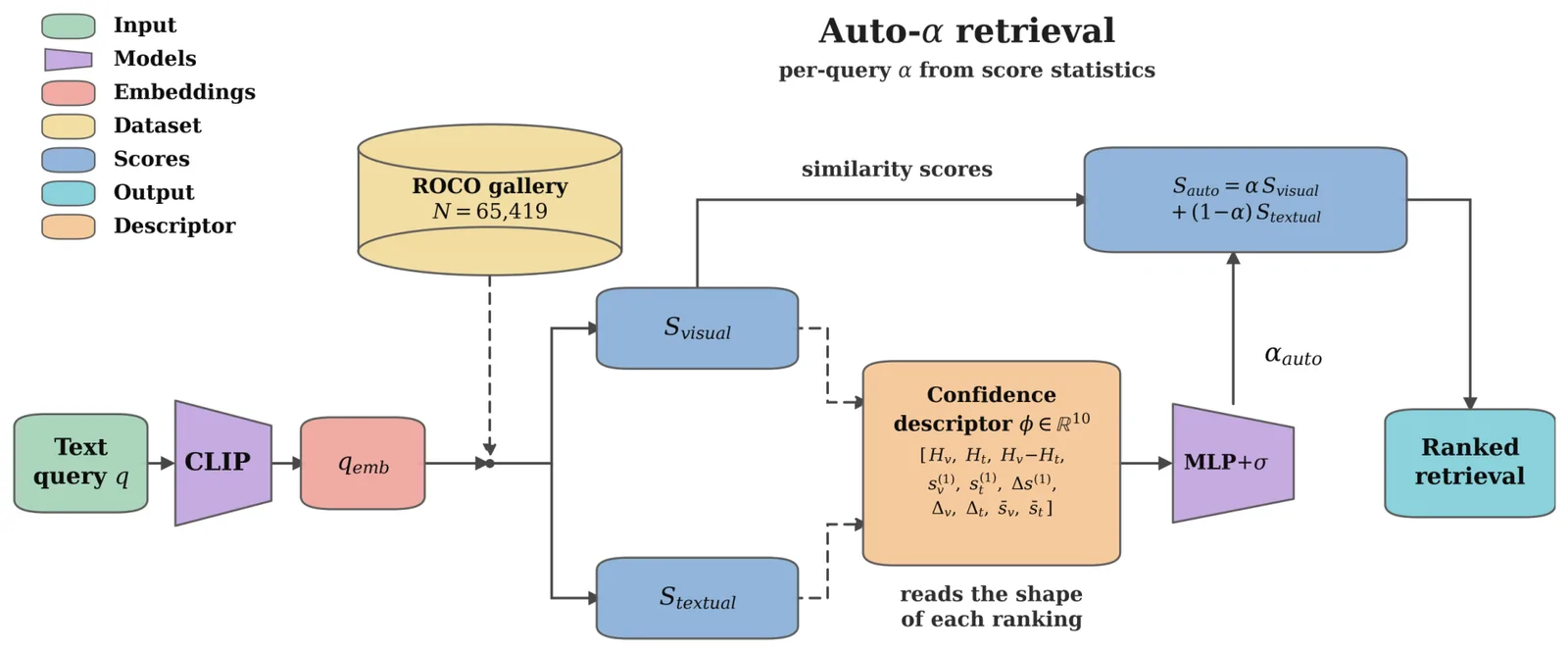
Pipeline of the cross-modality retrieval, where each modality is weighted by a per-query weight (α) predicted automatically from the score distributions, instead of a fixed user-tuned hyperparameter.

As shown in Figure 4, this approach is based on the premise that the ideal balance between modalities depends on the shape of their respective retrieval score distributions. Therefore, this approach is based on the concept that, if one modality is highly confident, meaning its top retrieved item has a higher similarity score than the rest of the gallery, that modality should receive a higher weight.

Given a query, we first compute its textual and visual similarity scores, $S_{t}$ and $S_{v}$, against all *N* gallery items. For each modality (m∈{t=textual,v=visual}), we sort these scores in descending order, $s_{m}^{\left(1\right)}\geq s_{m}^{\left(2\right)}\geq \cdots \geq s_{m}^{\left(N\right)}$, and define $p_{m}=\mathrm{softmax}\left({\mathrm{top}}_{k}\left(S_{m}\right)/\tau \right)$ as the probability distribution over the top k=100 scores, using a temperature τ=0.05. Both hyperparameters were selected by grid search with these values yielding the best Recall on the heterogeneous query pool.

We then extract three metrics for each modality to quantify this confidence, as defined collectively in Equation (3): The entropy ($H_{m}$) measures how peaked the ranking distribution is, where a lower entropy indicates higher confidence. The margin ($\Delta_{m}$) evaluates the score difference between the first and second ranked items. Finally, ${\bar{s}}_{m}$ represents the average score of the top 10 matches.

(3) $$H_{m}=-\sum_{i}p_{m,i}\log p_{m,i},\;\Delta_{m}=s_{m}^{\left(1\right)}-s_{m}^{\left(2\right)},\;{\bar{s}}_{m}=\frac{1}{10}\sum_{i=1}^{10}s_{m}^{\left(i\right)}$$

Using these statistics alongside simpler features, we construct a 10-dimensional descriptor, defined in Equation (4), that captures the confidence distributions of both modalities.

(4) $$\phi =\left[\;H_{v},\;H_{t},\;H_{v}-H_{t},\;s_{v}^{\left(1\right)},\;s_{t}^{\left(1\right)},\;s_{v}^{\left(1\right)}-s_{t}^{\left(1\right)},\;\Delta_{v},\;\Delta_{t},\;{\bar{s}}_{v},\;{\bar{s}}_{t}\;\right]$$

This descriptor is then standardized to have zero mean and unit variance, and then passed through a Multilayer Perceptron (MLP) consisting of a single hidden layer with 8 units (selected empirically through a grid search) and a ReLU activation function. The output is bounded by a sigmoid function (σ) to yield the predicted weight (see Equation (5)).

(5) $$\alpha_{auto}=\sigma \;\left(\mathrm{MLP}(\phi )\right)\in \left(0,1\right)$$

The final similarity score used for retrieval is the combination of both modalities, weighted by this predicted query-specific coefficient, as detailed in Equation (6).

(6) $$S_{auto}=\left(1-\alpha_{auto}\right)\;S_{t}+\alpha_{auto}\;S_{v}$$

The MLP is trained to rank the correct image first. To achieve this, following Equation (7), the network learns to weight the modalities by minimizing the cross-entropy loss (L) of the temperature-scaled fused scores, where the scaling factor β was determined via grid search:

(7) $$L=-\log\frac{\exp(\beta \;S_{auto}\left[y\right])}{\sum_{j=1}^{N}\exp\left(\beta \;S_{auto}\left[j\right]\right)},\;\beta =20$$

Since Auto-α requires training, we adopt a 5-fold cross-validation protocol: the network is trained on four folds and evaluated on the held-out fold, so that every query is scored by a model that never saw it during training. The standardization statistics for the descriptor are estimated exclusively on the training folds. Because both the fold partition and the network initialisation are stochastic, we repeat the whole procedure with five random seeds and report the mean ± standard deviation across repetitions.

Finally, it is important to note that the two approaches (Section 4.2.2 and Section 4.2.3) are complementary rather than alternative. When students are aware that what they are describing is purely visual or purely textual, they can set α directly to the value that suits their query. The purpose of Auto-α is for queries that combine clinical terminology with visual description, where the appropriate balance is not evident even to the person writing the query. Both mechanisms remain available in the deployed platform.

### 4.3. Evaluation Procedure

To evaluate this modality weighting approach, we used the training set of the ROCO dataset as the gallery and randomly selected 200 target pairs. From these pairs, we created four distinct evaluation sets based on the primary source of evidence used to generate the queries; the same target images are used in the three homogeneous sets, so that the query-generation regime is the only variable that changes:

- Caption only: a paraphrase of the ground-truth caption, with the image hidden from the generator (an LLM), representing purely textual evidence. This simulates queries from users employing technical language without providing visual details.
- Image only: a description synthesized exclusively from the image, with no access to the caption, representing purely visual evidence. This simulates queries from users focusing heavily on visual characteristics.
- Caption + Image: a description grounded in both the image and its caption, representing mixed evidence. This acts as a middle ground to simulate hybrid queries.
- Mixed: the union of the three sets above. This represents the most realistic scenario, accommodating users who might alternate between text-heavy and visual-heavy queries.

For the evaluation of the mixed set, the folds were partitioned by target image to prevent cross-modality leakage of target identities, so that queries pointing to the same image never fall on both sides of the split.

#### 4.3.1. Comparison Baselines

We compare Auto-α against nine alternatives from four different families.

- Single-modality: retrieval using only captions (α=0) or only images (α=1), the degenerate cases of Equation (2).
- Fixed-weight fusion: a single α grid-searched in steps of 0.05, tuned either on the whole mixed set or separately on each query type; the latter requires knowing the query type in advance and is reported for reference only, as it is not deployable.
- Parameter-free fusion: CombSUM with per-query *z*-score normalisation [64], and Reciprocal Rank Fusion [65], which fuses by position, $\sum_{m}1/\left(k+r_{m}\right)$ and is therefore invariant to the modality gap; we keep k=60, the value used in the original work.
- Query-adaptive fusion: a confidence-gating control that assigns all weight to the modality whose top-1 score deviates most from its own top-*k* distribution; an adapted implementation of Query-Adaptive Late Fusion [28], which estimates modality reliability from the area between the sorted score curve and a reference curve; and an entropy-based scheme [66] that weights each modality by its inverse normalised Shannon entropy. Since we do not have access to the external reference collections used by the authors, its reference curves are built from gallery probes with their true match removed. We additionally report a per-query oracle that selects the best grid α for each query using the ground-truth label; it is not a method but an upper bound on what any per-query weighting scheme could achieve.

#### 4.3.2. Ablation Protocol

To quantify the contribution of each component separately, we evaluate four controlled variants, each isolating a single factor: first, the text branch alone with visual features removed; second, the visual branch alone with text input removed; third, both branches with the MLP weight predictor replaced by entropy weighting, which separates the benefit of learning the weight from the benefit of merely making it query-adaptive; and finally the full model. Top-1 Recall is reported for each variant on all four query types.

#### 4.3.3. Unified Configuration and Statistical Protocol

All methods are evaluated under an identical configuration: the same CLIP encoder, the same 65,419-image gallery, the same queries and ground-truth targets, the same GPU and float32 precision throughout. The only difference between compared methods is how α is computed. Variability is reported as the standard deviation over five random seeds, which affects only the two methods whose behaviour depends on a random draw: Auto-α, whose seed controls both the cross-validation fold partition and the network initialisation, and Query-Adaptive Late Fusion, whose seed selects the probes forming its reference codebook. All remaining methods are deterministic functions of the similarity scores and therefore carry no seed variance.

### 4.4. Synthesis Performance

Traditional diffusion models, like our baseline model Prompt2MedImage [22], generate images by slowly removing noise over several iterative steps. Therefore, creating synthetic images using diffusion models may be computationally expensive, specifically when the hardware used is CPUs.

To solve this limitation, we integrated a Latent Consistency Model Low-Rank Adaptation (LCM-LoRA), that aims to reduce the number of iterations required to generate the synthetic images. Instead of training a new model from scratch, LCM-LoRA acts as a lightweight mathematical “plug-in”, specifically, we used lcm-lora-sdv1-5 [67], that teaches our existing medical model how to predict the final image in a reduced number of steps.

To adapt the architecture of the model, we first replaced the standard mathematical solver with an LCMScheduler [47], which allows the model to take larger steps during the denoising process. Second, instead of keeping the base model and the LCM-LoRA adapter as separate pieces, we permanently fused their weights together to prevent the system from wasting computational resources communicating between separate components during image generation.

To compare the standard diffusion model and the LCM-LoRA approach, we conducted a hyperparameter grid search to determine the optimal values for the number of inference steps and the guidance scale. While the number of inference steps represents the iterations used to process the image, the guidance parameter controls how closely the model follows the text prompt, with higher values forcing stricter adherence to the query text. The goal was to find the configuration that best balances image quality and generation time for each method. The optimal settings were identified as the combination that produced the highest CLIP score relative to the text query. If multiple configurations yielded similar scores, we prioritized the one with the lower computational cost. Optimal configuration is shown in Table 1.

Once the best configuration was established, we developed an evaluation using 200 medical queries to evaluate the efficiency of both approaches. For each query, we generated two images, one using the diffusion model and the other using the LCM-LoRA approach. During the generation of these images, we saved the latency in seconds to generate each image and the computational cost (FLOPs) used per generation. Both metrics follow how acceleration is characterised in the consistency-model literature, where speedup is expressed as the reduction in denoising steps and therefore in network evaluations per image [45,47]. We convert that count into FLOPs, which stays comparable across devices, and report wall-clock latency alongside it because a claim about deployability on free CPU infrastructure can only be demonstrated in measured seconds.

In order to evaluate if this acceleration process decreased the quality of the generated images, we evaluated the outputs using the CLIP model. For the 200 queries, we used this model to obtain its latent space representation, subsequently repeating the process for the two synthetic images generated per query to obtain their corresponding embeddings. Then, we compared on the one hand the embedding of the text query against the embedding of the synthetic image generated using the standard diffusion model and calculated the cosine similarity between them; then, we repeated the same operation but instead of using the synthetic image generated with the standard diffusion model, we used the synthetic image created with the LCM-LoRA pipeline. By comparing the difference between both CLIP scores, we can evaluate the variation in the generation of synthetic images.

While metrics like the CLIP score may establish a mathematical baseline, they cannot assess true anatomical correctness. Therefore, to obtain clinical validation, we conducted a blinded expert evaluation in collaboration with a hospital radiology service. A radiologist reviewed the 200 text queries alongside their corresponding generated image pairs, comparing the standard diffusion model against the LCM-LoRA approach. To prevent any grading bias, the presentation order of the images was randomized for each query. The radiologist was tasked with scoring the semantic and clinical accuracy of each synthetic image relative to the prompt on a scale from 0 to 100.

Following data collection, we performed a statistical analysis to evaluate the trade-off between computational efficiency and clinical quality. We quantified computational performance by measuring generation latency (in seconds) and computational cost (in TFLOPs), while generation quality was assessed using both the CLIP score and the score given by the radiologist.

Since our dataset consisted of paired samples, we applied the Wilcoxon signed-rank test [68] to determine if the differences in clinical quality and CLIP scores between the two methods were statistically significant. Additionally, we used Cohen’s d [69] to measure the effect size of these differences. Finally, to investigate whether the automated CLIP metric serves as a reliable proxy for human medical judgment, we calculated the Pearson correlation coefficient [70] to evaluate the relationship between the automated CLIP scores and the subjective expert evaluations provided by the radiologist.

**Figure 5:**
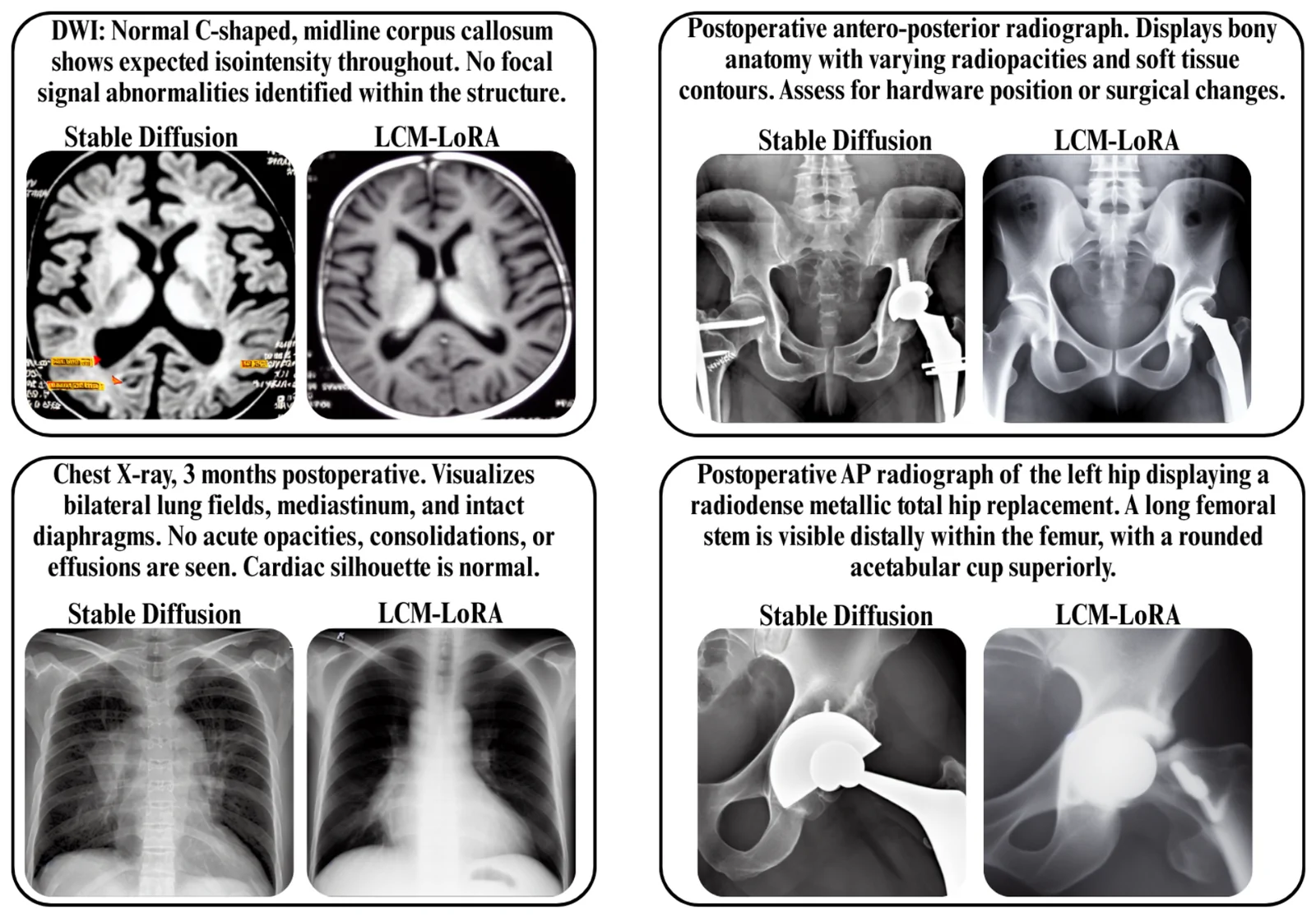
Examples of synthetic images generated using the same query but different approaches: first, the standard diffusion model, and then, the LCM-LoRA pipeline.

Figure 5 displays four examples of synthetically generated images based on a specific query. While both approaches maintain a highly similar anatomical structure, notable differences emerge in image intensity. Specifically, the Stable Diffusion model is capable of generating finer details, such as the burned-in text typically found in clinical annotations, or a more realistic prosthesis in the right-hand image. The bottom row of Figure 5 shows the cases where one of the methods struggles to represent the queried concept. For the chest radiograph on the left, LCM-LoRA renders perfectly the lung fields, mediastinum, and diaphragms described in the query, whereas Stable Diffusion does not, according to the expert radiologist who annotated the images. In the images on the right that show the total hip replacement, Stable Diffusion places a recognizable femoral stem and acetabular cup, while LCM-LoRA struggles to ideally reproduce the described query.

### 4.5. Auditing System

Frequently, retrieval systems lack a feedback mechanism to warn the user when a retrieved image does not match their query. To address this, we implemented an automated auditing system. The concept is that once an image is retrieved based on a user query, an LLM acts as a “second opinion” to generate a confidence score on the retrieved image.

In order to validate this automated auditor, we designed an experiment where we built a ground-truth dataset of 200 query-image pairs. Specifically, the dataset was divided into three categories. First, we created 50 positive pairs, representing perfect matches where the query is the actual ground-truth caption of the image. Then, we generated 50 negative pairs by pairing an image with a random caption from a completely unrelated pair in the dataset. Finally, to simulate intermediate scenarios where a retrieval is only partially accurate, we generated 100 intermediate pairs. To do so, the original caption was synthetically altered using an LLM to change its semantic content; specifically, we divided this subset into three sections of pairs: 33 pairs with a 25% modification, 33 pairs with a 50% modification, and 34 pairs with a 75% modification. A total of 200 pairs was chosen based on the trade-off between having enough samples for each subset and keeping a manageable number for expert annotation, since every pair had to be scored individually by a single radiologist, a well-documented bottleneck in medical imaging research [71].

To test the auditing capabilities of different AI models, each of these 200 query-image pairs was passed through three state-of-the-art lightweight multimodal LLMs: Gemini 2.5 Flash [72], Pixtral 12B [73], and Llama 4 Scout 17B [74]. Each model received the query and the corresponding retrieved image and was instructed to evaluate this match. To ensure a fair evaluation, all three models were queried using the exact same prompt.

Finally, in order to establish a clinical baseline, we replicated this evaluation setup with an expert radiologist who received the same 200 pairs and was asked to evaluate their relevance on a 0 to 100 scale. By aligning the AI-generated scores alongside the ground-truth evaluations of the radiologist, we analyzed correlations to determine the extent to which these multimodal LLMs can imitate human clinical judgment in an auditing task.

To evaluate the performance of the LLMs as clinical auditors, we compared the scores they generated against the evaluations provided by the radiologist. We assessed linear correlation and absolute agreement by calculating Pearson’s correlation coefficient (with 95% confidence intervals) and the Intraclass Correlation Coefficient [75] (specifically the two-way random-effects model, ICC(2,1)), alongside the Mean Absolute Error (MAE). Then, to detect any systematic scoring bias, we conducted a Bland-Altman analysis [76] to calculate the mean bias. Finally, to determine if one LLM significantly outperformed the others in aligning with the radiologist’s assessments, we applied the Steiger test [77].

### 4.6. User Experience

To enhance the accessibility we deployed the system on Hugging Face with an easy, ready-to-use interface that does not require any installation. We decided to use this platform since it enables access to a free CPU with 16 GB of RAM, which is enough capacity to run the platform. Additionally, we also deployed the platform on GitHub [78] to download and set it up locally, where the use of a GPU could speed up all processes.

**Figure 6:**
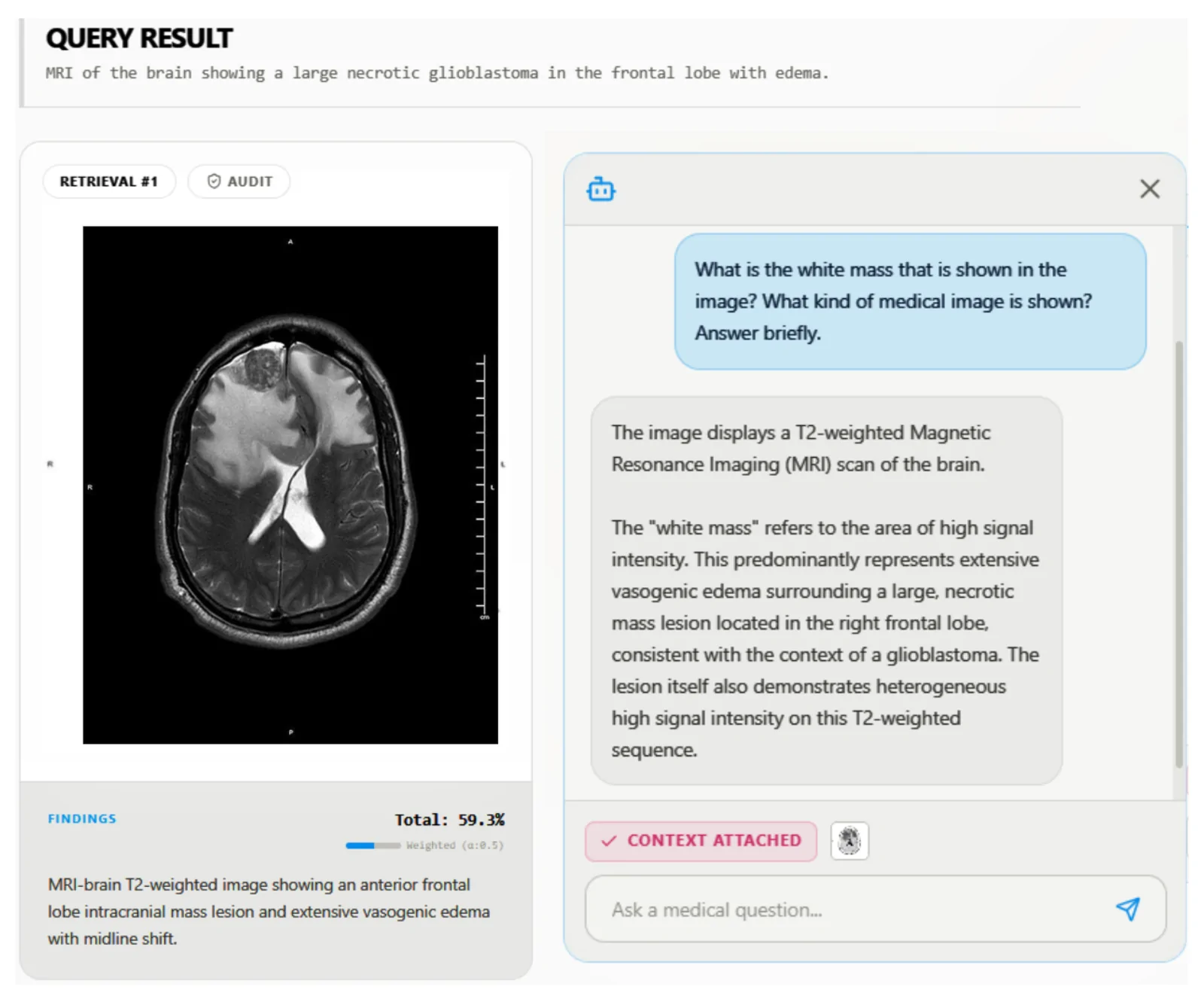
Example of the chatbot acting as an educator capable of answering questions related to the retrieved medical images.

**Figure 7:**
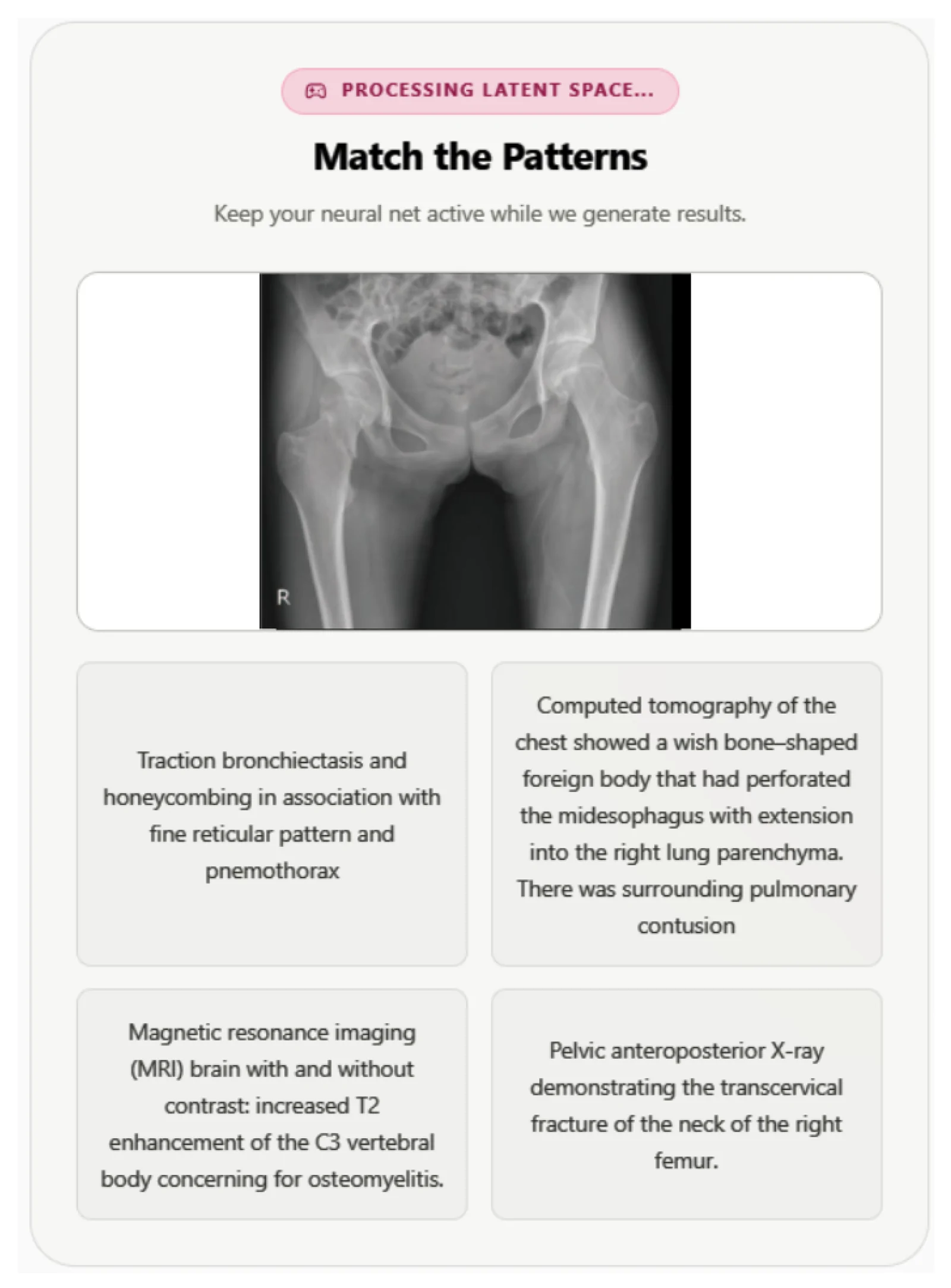
Example of an educational quiz presented during the generation of synthetic images.

To further enrich the system we introduced several secondary interactive enhancements:

- We integrated multilingual support through an automatic translation module, allowing students to input queries in their native languages to ease the learning process. To do so, an LLM translates the input query of the user into English. The system then processes the query in this central language. Finally, all the explanations, descriptions, and other texts are translated back into the native language of the user.
- To transform the platform into an interactive learning environment, as shown in Figure 6, we added a contextual chatbot that acts as a virtual teacher. This allows students to ask questions related to the retrieved images or the medical content of their queries.
- Finally, to encourage active learning, as illustrated in Figure 7 we included a quiz tool that is displayed while the student waits for the generation of synthetic images. Although these waiting times are not extremely long, our goal is to maximize the learning time. These quiz questions ask the student to match a caption from the ROCO database with its corresponding image, or vice versa, matching a displayed image to its correct caption.

## 5. Results

In this Section we present the results obtained from the evaluation of the validated parts of the pipeline.

### 5.1. Retrieval

We evaluated the two cross-modality retrieval approaches on the four query sets described in Section 4.3. We first analyze the behaviour of the fixed-weight fusion as a function of α, then compare the best fixed α against the learned, per-query Auto-α, and finally position both against nine baselines under a unified configuration and isolate the contribution of each component through a controlled ablation.

**Figure 8:**
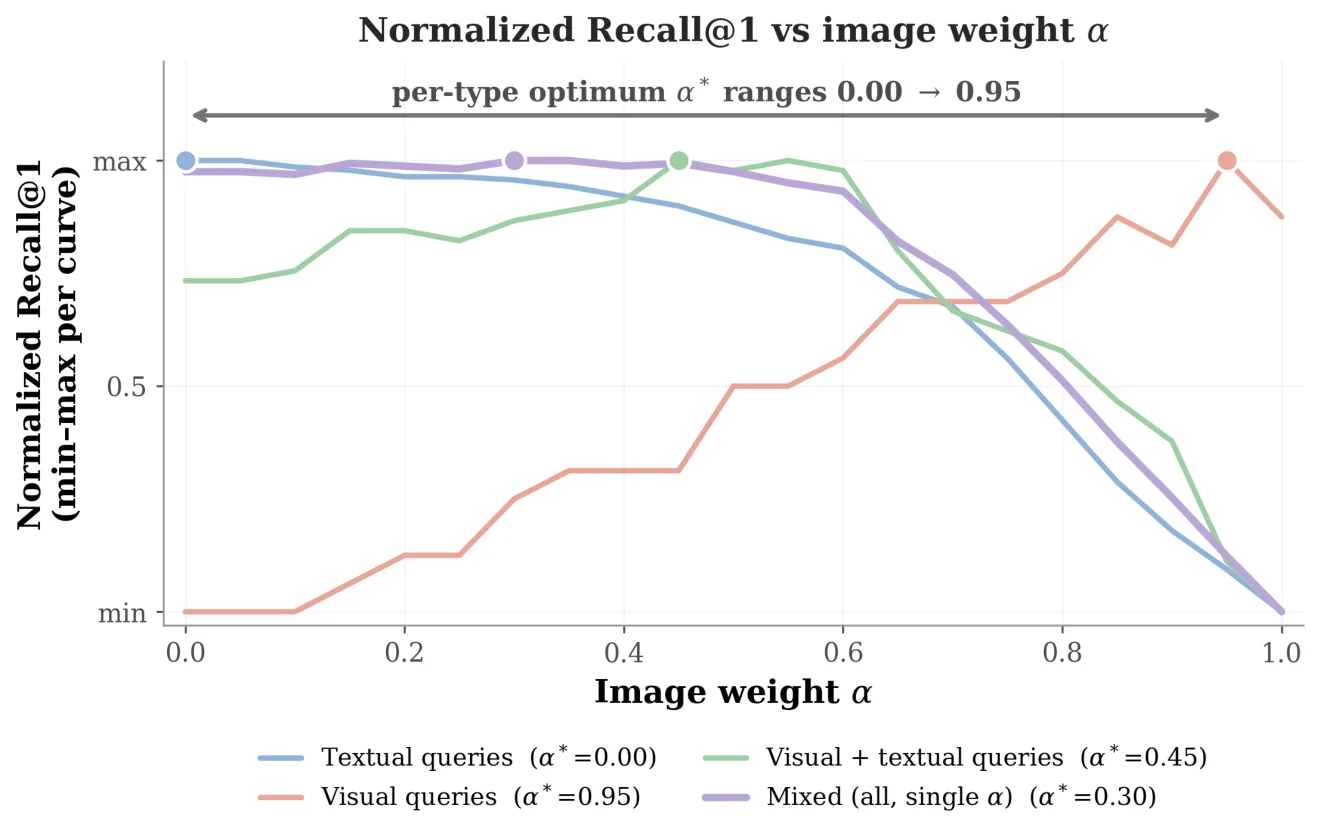
Top-1 Recall as a function of the fusion weight α for each query subset, min–max normalized per curve so that the shapes remain comparable despite differing absolute levels. The optimal α shifts with the query type, from 0.00 for caption-derived queries to 0.95 for image-derived ones.

The baseline α was a single optimized value for the complete subset that maximized Top-1 Recall. In Figure 8, we show how different values of the parameter α affect retrieval performance for the four subsets described in Section 4.3. Performance is measured as Top-1 Recall and min–max normalized within each subset so that every curve ranges from 0 to 1, making the curve shapes comparable despite differing absolute Top-1 Recall levels. The optimal weights ($\alpha^{*}$) identified for each category are summarized in Table 2.

As shown, the value of α that maximizes Top-1 Recall shifts significantly according to the query type. This suggests that the optimal fusion weight α strongly depends on the nature of the query, highlighting the importance of tuning this parameter during image retrieval based on the user’s input modality.

**Figure 9:**
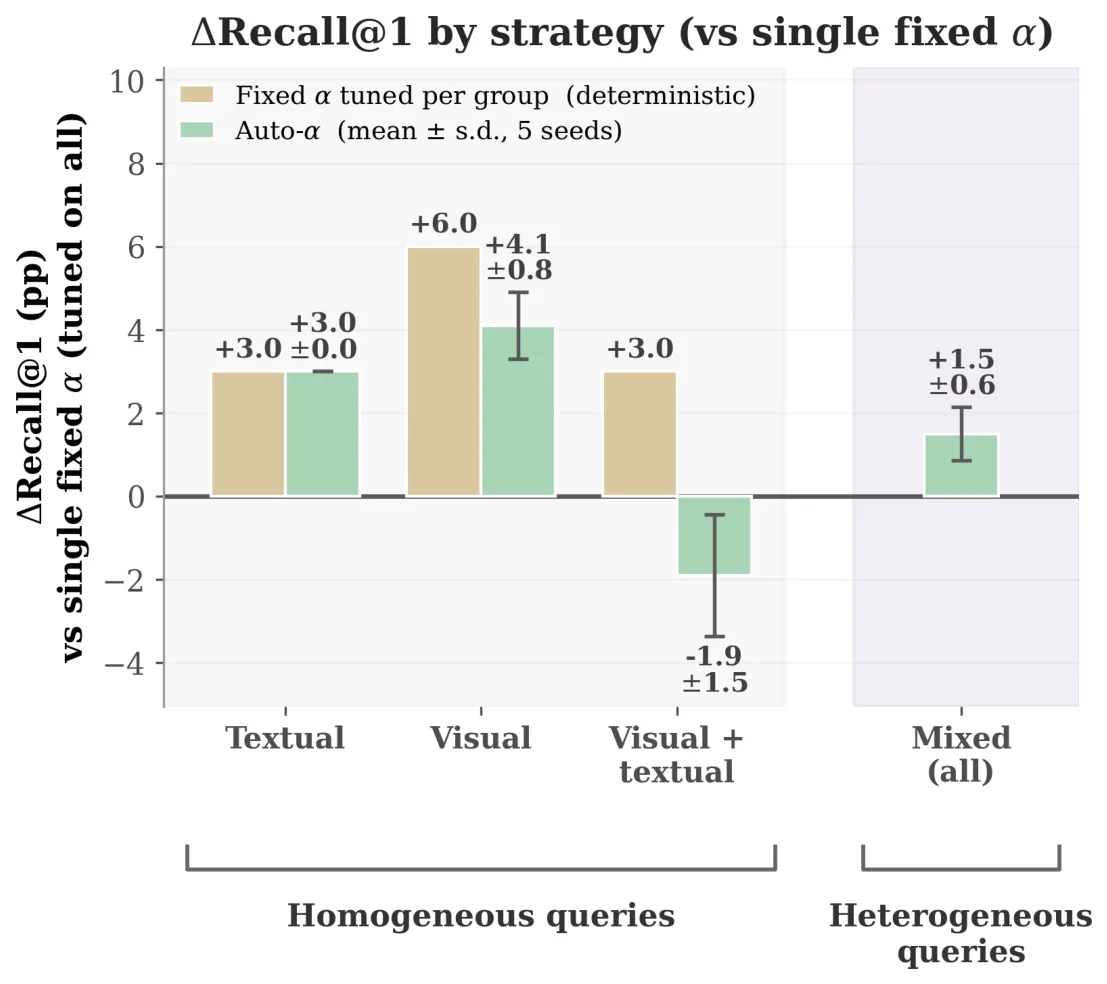
ΔTop-1 Recall relative to a single fixed α tuned on the whole dataset, for the per-group best fixed α (yellow) and Auto-α (green). No fixed-α bar is shown on the mixed set: its best fixed α is by definition the one tuned over the whole dataset, so its difference would be zero.

The Figure 9 compares the best fixed α (a single value tuned for each subset) against the Auto-α approach. Within each homogeneous set, a single fixed weight achieves the best performance. For Caption-only queries, Auto-α matches the performance of the fixed α, as the MLP learns to consistently predict α≈0. On the other two homogeneous subsets, Auto-α achieves a lower performance than using a fixed α. For the heterogeneous set, no fixed-α (yellow) bar is shown: its best fixed α is, by definition, the single α tuned over the whole dataset, so its difference would be 0%. This indicates that the adaptive weight for each query is beneficial when the queries are heterogeneous, which represents the most realistic deployment scenario.

It is also important to note that the best fixed values of α were obtained for specific, isolated sets of queries. Real-life queries, however, are rarely this strictly categorized; rather, they contain a mix of different types of information, further highlighting the relevance of the Auto-α approach. A closer look at the predicted weights explains where the advantage comes from. The optimal α moves from 0.00 to 0.45 to 0.95 across the three homogeneous sets, and Auto-α follows it: its mean prediction moves from 0.001 to 0.138 to 0.675, spanning two thirds of the weight range. The gain is therefore not a by-product of averaging two modalities, but of identifying which one to trust. Auto-α nevertheless under-shoots the optimum consistently, which is coherent with the modality gap between images and text in the dataset and leaves room for improvement.

We compared our query-specific Auto-α mechanism against nine baselines, ranging from single-modality retrieval and fixed-weight fusion to parameter-free rank fusion and query-adaptive schemes. To make the comparison fair, every method was executed in a single pass of the same script, so all of them share the CLIP encoder, the 65,419-image gallery, the queries and their ground-truth targets, the hardware and float32 precision; the only difference between rows is how α is obtained. Table 3 reports the resulting comparison.

Table 3 shows that no fixed strategy is robust across query types. The text-only branch is the best option for caption-derived queries but collapses to 4.50% when the query describes what is seen in the image, and the visual-only branch fails symmetrically. Reciprocal Rank Fusion, which combines by position and ignores the score values altogether, weights both modalities equally by construction and therefore drops to 18.83% on the mixed set. Auto-α is the only method that stays competitive on all four sets and the best one on the mixed set, at the cost of losing to the per-type fixed α on the two image-involving subsets.

On the mixed set, however, Auto-α attains 38.83±0.64% Top-1 Recall, the highest of all evaluated methods. It improves over every one of the nine baselines: by 28.50 and 2.17 pp over the visual-only and caption-only branches, by 20.00 pp over Reciprocal Rank Fusion, 4.07 pp over Query-Adaptive Late Fusion and 3.00 pp over CombSUM. The margin narrows against the closest competitors, reaching +1.83 pp over confidence gating, +1.50 pp over the tuned fixed α and +1.33 pp over entropy-based weighting. These three are the only methods that come within two points, and none of them adjusts α in a way that follows the per-regime optimum: the fixed weight cannot adapt at all, confidence gating can only hand the query to one modality outright, and entropy-based weighting varies α over a range of 0.14 against the 0.95 spanned by the optimal values. Auto-α covers 0.67 of that range, which is where its advantage on the mixed set comes from.

Query-Adaptive Late Fusion performs below the text-only branch in our setting. Its weights concentrate around 0.48 regardless of the query type, so it degenerates into an approximately fixed α=0.5. This is consistent with its design assumption, since it compares the shape of two score curves expected to be commensurable, whereas here the visual curve is systematically flatter than the textual one because of the modality gap, and the difference in shape carries little query-specific information.

Regarding the ablation study, it follows a controlled-variable design where each variant changes one factor at a time and everything else is held fixed. Beyond the three control groups, we include an intermediate reference in which both branches are fused with a fixed α; without it, the benefit of combining the two modalities cannot be separated from the benefit of adapting the weight.

As we can see in Table 4, removing either branch collapses performance on the opposite query type, which is the clearest evidence that both modalities are needed. The text-only variant falls to 4.50% on image-derived queries and the visual-only variant to 7.50% on caption-derived ones. The cumulative decomposition on the mixed set, shown in the last column of Table 4, attributes the gain to each component: fusing both branches with a fixed weight adds +0.67 pp over the better single branch, making that weight query-adaptive with a fixed entropy rule adds a further +0.17 pp, and learning it with the MLP adds +1.33 pp. Most of the improvement therefore comes from learning the weight rather than from merely adapting it, since a rule that is equally query-adaptive but not learned contributes almost nothing.

**Figure 10:**
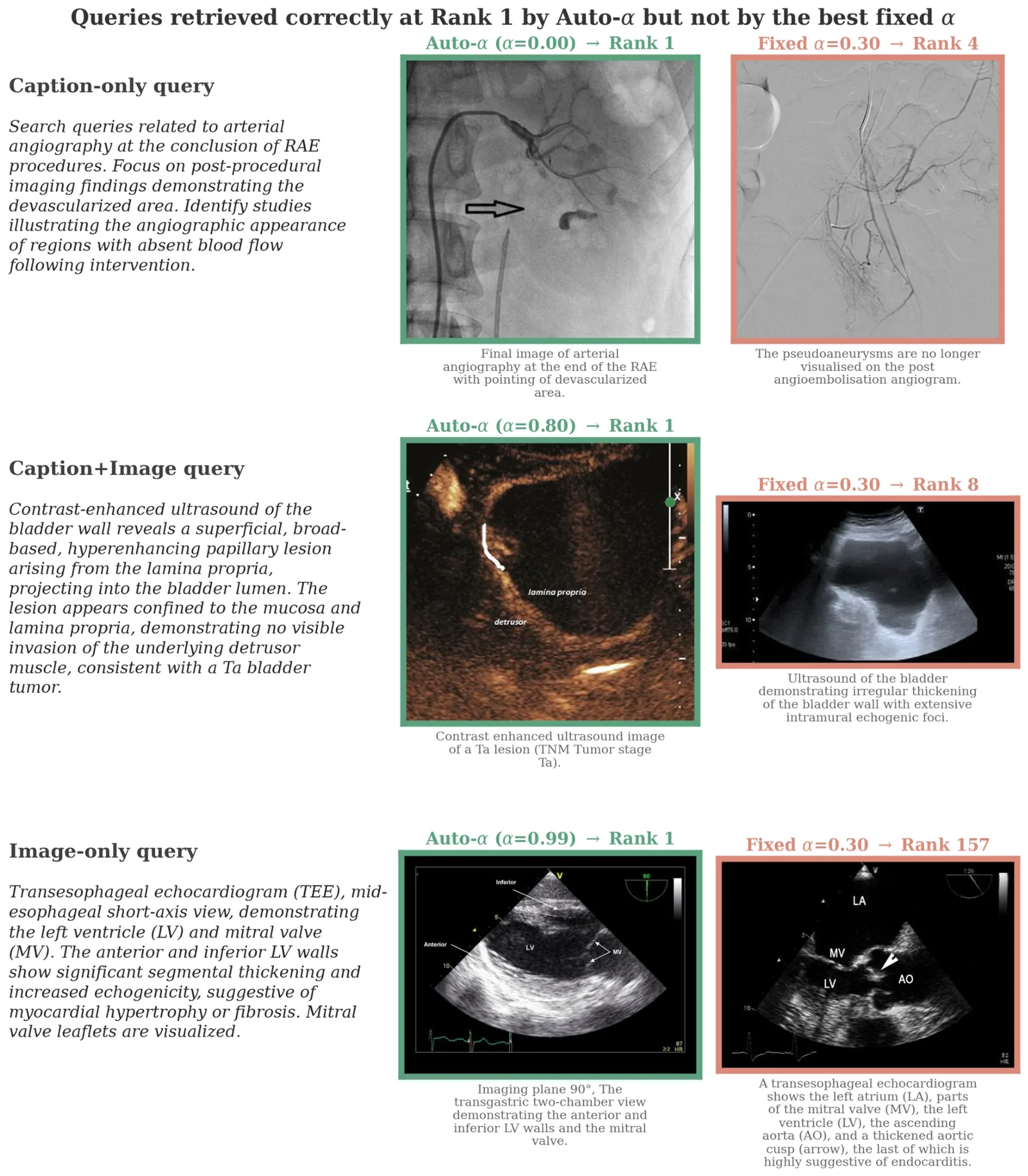
Examples of improved retrieval using Auto-α. Each case shows the top-ranked image by Auto-α and the new rank assigned to the baseline’s top choice.

To observe examples where the Auto-α approach provides clear benefits, Figure 10 presents three cases, one from each of the three homogeneous subsets, where this method accurately reranks the images. For each case, we show the query, the first-ranked image retrieved by the Auto-α approach, and the rank assigned by Auto-α to the image that the fixed α baseline (a single value for the complete set) had originally ranked first. These examples suggest that the benefit of an adaptive weight is highly valuable on miscalibrated values for α, a risk that students may produce during the use of the system.

### 5.2. Synthesis

To evaluate the trade-off between computational efficiency and clinical quality, we analyzed 200 paired synthetic images. The computational performance results are summarized in Table 5. The LCM-LoRA optimization proved to be significantly more efficient, reducing the computational cost by a factor of 12.5× and lowering latency on both GPU and CPU environments.

The speedup is bounded by the denoising loop alone. Standard sampling performs 25 steps with classifier-free guidance, that is 50 network evaluations per image, whereas LCM-LoRA performs 4 steps with a single evaluation each; the range therefore runs from 1×, if runtime were dominated by costs that do not depend on the step count, to 12.5×, if it were dominated entirely by denoising. The measured 4× on GPU and 2.7× on CPU fall at 32% and 22% of that upper bound. The adapter therefore removes essentially all of the denoising cost, and what remains is the part of the pipeline it does not touch.

Regarding clinical quality, the blinded radiologist evaluation is detailed in Table 6, which demonstrates that the standard diffusion model achieves higher clinical quality (+8.66 pp) in the generated images compared to those produced using LCM-LoRA. A Wilcoxon signed-rank test confirmed that the difference in perceived quality was statistically significant (p=0.0004). However, the calculated Cohen’s d for paired samples was 0.248, suggesting a small effect size. This demonstrates that while the standard model produces slightly more accurate anatomical representations, the loss of clinical quality in the LCM-LoRA approach is compensated by its gains in computational speed and efficiency.

Finally, we analyzed the relationship between the CLIP score and human medical perception. The pooled data analysis revealed a statistically significant, but weak, positive Pearson correlation (r=0.237,p<0.001) between the CLIP scores and the clinical scores of the radiologist. This suggests that while CLIP provides a mathematically valid baseline for text-image alignment, it is not an ideal metric for clinical correctness. These results pave the way for applying the auditing system to synthetic images in order to assess their quality.

### 5.3. Audit

The statistical analysis was based on the 200 evaluated samples which, as explained in Section 4.5, comprise real pairs from the database, mistaken combinations created by randomly associating images with random captions from the ROCO database, and modified instances. Our evaluation of the auditing module yielded two primary findings, comprehensively detailed in Table 7:

- Gemini significantly outperformed the other tested LLMs as a clinical auditor. It proved to be the most accurate model, achieving the highest linear correlation with the human expert (Pearson r=0.805) and the strongest absolute agreement (ICC = 0.806). Furthermore, the Steiger test [77] confirmed that the superiority of Gemini over both Pixtral and Llama 4 was statistically significant (p<0.001).
- Using an LLM corrects the overestimation inherent to the CLIP metric. While the CLIP score correlates with human judgment, the Bland-Altman analysis suggests that it systematically overestimates retrieval quality, presenting a high mean bias of +0.237 and a Mean Absolute Error of 0.281. In contrast, Gemini effectively eliminated this systematic error: it reduced the mean bias to near zero (−0.006) and cut the absolute error in half (MAE = 0.143), proving to be a more reliable proxy for human scoring.

**Figure 11:**
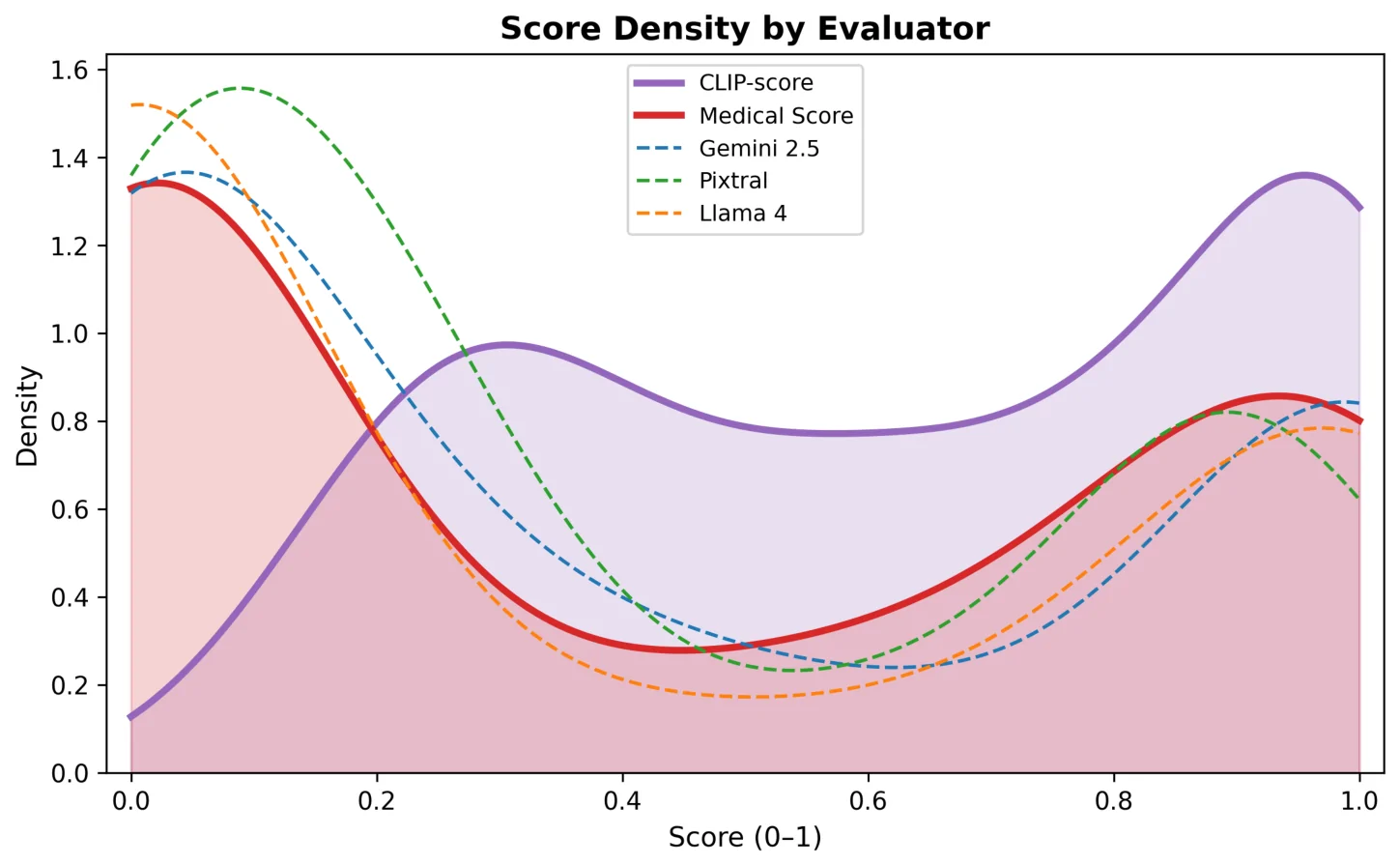
Kernel density estimate of the scores assigned by each evaluator across the 200 audit pairs. Solid lines (CLIP score and Medical Score) are shown with shaded fills; LLM evaluators are shown as dashed lines.

As visually supported by Figure 11, which presents the score density distribution for all evaluators, there is a noticeable behavioral gap between the CLIP score and human judgment. While CLIP score overestimates retrieval quality, clustering its scores around the center of the distribution, the medical score provided by the radiologist demonstrates a bimodal distribution, penalizing incorrect retrievals (peaks near 0) and rewarding perfect matches (peaks near 1). Notably, the dashed lines representing the multimodal LLMs closely imitate this bimodal distribution, confirming that the AI auditors evaluate clinical alignment much more naturally than standard latent-space similarities.

It is interesting to note that Gemini deviates from the radiologist by 0.143 on average against 0.197 for Llama, an error 38% larger. Both auditors nonetheless improve on the CLIP baseline in agreement, and Llama does so despite correlating with the expert less closely than CLIP itself (r=0.679 against 0.780). Globally, another finding is that CLIP ranks retrievals reasonably well but places them on the wrong scale, while the LLM auditors reproduce the values that the expert would have assigned.

A strict comparison of auditing latency is not meaningful here, since all three models are queried through their APIs providers and the observed response time reflects queueing and network transport rather than the model itself.

## 6. Discussion

The integration of artificial intelligence into medical education requires tools that are not only technologically advanced but also computationally accessible and clinically reliable. In this extended framework, we addressed the core limitations of the original MIRAGE system by introducing adaptive multimodal retrieval, accelerating generative pipelines, deploying an automated clinical auditor, and ensuring their reliability through expert radiologist validation, among other novelties.

Our findings regarding the Auto-α retrieval mechanism highlight that, in a medical context, user queries are heterogeneous, and the way the query is expressed is directly related to the ideal weight to obtain the retrieved image. While a fixed fusion weight performs optimally in homogeneous scenarios (such as purely textual or purely visual queries), it struggles in mixed contexts. The consistent gain of Auto-α on the mixed query set, together with the fact that it is the only method whose predicted weight follows the optimum closely across query types, suggests that dynamically predicting the modality weight per query is essential for real-world deployments, where students may alternate between clinical terminology and visual descriptions. By automatically calculating the importance of each modality for each query, Auto-α protects the user from miscalibrated manual weighting.

Furthermore, the integration of the LCM-LoRA optimization presents a favorable trade-off between computational efficiency and clinical quality. The deployment of generative AI in educational tools is often hindered by prohibitive hardware requirements. Our results show a 12.5× reduction in computational cost and a massive decrease in latency (from over 6 min to roughly 2 min on a CPU). While the expert radiologist evaluation noted a statistically significant but small drop in anatomical precision (Cohen’s d=0.248), this can be considered a reasonable compromise given that it enables the diffusion model to run effectively on free, CPU-powered cloud environments like Hugging Face.

The most impactful finding of this study, summarized in Table 7, is the validation of multimodal LLMs as clinical auditors. Although the CLIP score correlates linearly with human judgment (Pearson r = 0.780), the Bland–Altman analysis reveals poor agreement (ICC = 0.609) and a large bias (+0.237): the metric overestimates text–image alignment and fails to capture clinical relevance. By deploying Gemini 2.5 Flash as a “second opinion,” the system mirrors the bimodal scoring behavior of a human expert and removes this systematic bias: Gemini reduces the mean bias to near zero (−0.006), halves the absolute error (MAE = 0.143) and raises the absolute agreement to ICC = 0.806, while matching the expert with a Pearson correlation of 0.805. This auditor provides students with immediate feedback, warning them when a retrieved image may not depict their query.

Despite these promising results, this study presents certain limitations. Regarding retrieval, the gap to the per-query oracle (46.17% vs. 38.83%) indicates that a considerable margin remains for better weight prediction. The clinical validation of both the synthetic images and the auditing module relied on the evaluation of 200 query-image pairs by a single expert radiologist. While this provides a strong baseline, future work should involve a larger panel of medical experts to account for inter-observer variability.

Lastly, our experiments use a single dataset. ROCO is a broad one, since it is built from open-access medical publications: its images come from many different hospitals and cover a wide range of imaging types, body regions and conditions. Testing the system on a second dataset would still be useful, but we did not find one that aligns with the requirements of our project. We will revisit this as new resources become available.

Beyond that, our own results point to two different directions. First, the gap that separates Auto-α from the per-query oracle shows that most of the remaining work lies in the weight predictor. Second, on the generation side, the denoising loop is no longer the bottleneck, so any further acceleration will have to target the decoder rather than the number of sampling steps.

## 7. Conclusions

In this paper, we presented a multimodal educational platform designed for medical students. By extending our preliminary MIRAGE framework, we mitigated its previous computational bottlenecks:

First, the introduction of the Auto-α mechanism removed the need for the user to estimate the fusion weight on every search achieving the best Top-1 Recall among all evaluated fusion strategies on heterogeneous medical queries, and being the only one whose predicted weight adapts to the type of query.

Second, the integration of an LCM-LoRA adapter into the diffusion pipeline reduced inference latency and computational overhead, allowing synthetic medical image generation to run on accessible CPU hardware without sacrificing clinical value.

Finally, we demonstrated that state-of-the-art multimodal LLMs can act as clinical auditors that more faithfully reflect expert judgment than embedding-based metrics. Among the evaluated models, Gemini 2.5 Flash achieved the strongest agreement with the radiologist while reducing the overestimation bias inherent to the CLIP score. Pixtral 12B and Llama 4 Scout, both considerably smaller, ranked below Gemini but still surpassed the CLIP baseline in absolute agreement and error.

Coupled with new user experience features such as multilingual support and interactive quizzes, this open-access framework provides an interactive and reliable learning environment. Future research will focus on expanding the underlying image repository and conducting extensive user studies with medical students and educators to evaluate the long-term pedagogical impact of the platform in real-world academic settings.

## Figures and Tables

**Table 1:** Optimal parameters found via grid search.

	Stable Diffusion	LCM-LoRA
Steps	25	4
Guidance	12.5	1.5

**Table 2:** Optimal fusion weights ($\alpha^{*}$) per query type.

Query Subset	Optimal $\alpha^{*}$
Caption-only	0.00
Caption + Image	0.45
Image-only	0.95

**Table 3:** Integrated comparison of adaptive cross-modal fusion methods under a fully unified experimental configuration. Top-1 Recall (%) on the four query sets. The rule separates the deployable methods above from the two references below, which require information that is not available at query time; the best result per query set is shown in bold and is computed over the deployable methods only. Fixed global α is grid-tuned on the mixed set. A standard deviation is reported only for the methods that depend on a random data partition, and is computed over 5 random seeds: Auto-α, whose seed controls both the cross-validation fold partition and the network initialisation, and QALF, whose seed controls which probes form its reference codebook. All remaining methods are deterministic functions of the similarity scores, so no deviation applies to them. Best result per query type in bold. ^†^ Requires knowing the query type in advance; not deployable. ^‡^ Uses the ground-truth label; an unattainable upper bound.

Method	Caption Only	Caption + Image	Image Only	Mixed (All)
Text only (α=0)	**77.00**	28.50	4.50	36.67
Visual only (α=1)	7.50	12.00	**11.50**	10.33
Fixed global α	74.00	31.50	6.50	37.33
CombSUM + *z*-score [64]	64.50	**34.50**	8.50	35.83
Reciprocal Rank Fusion [65]	23.50	24.50	8.50	18.83
Confidence gating	73.00	29.00	9.00	37.00
Query-Adaptive Late Fusion [28]	64.80 ± 0.93	30.40 ± 1.46	9.10 ± 1.28	34.77 ± 0.74
Entropy-based weighting [66]	70.50	33.50	8.50	37.50
**Auto-α (ours)**	**77.00 ± 0.00**	29.60 ± 1.46	10.60 ± 0.80	**38.83 ± 0.64**
Fixed α per query type ^†^	77.00	34.50	12.50	37.33
Per-query oracle $\alpha^{\;‡}$	79.50	42.00	17.00	46.17

**Table 4:** Controlled ablation of Auto-α: each row isolates one component. Top-1 Recall (%) per query type. The last three rows use both branches and differ only in how α is set: a single grid-tuned value, a fixed entropy rule, or the learned MLP. The last column reports the contribution of the component added in that row, measured on the mixed set against the row above it; the chain starts from the better of the two single-modality branches, so neither of them has an increment. Only the learned model depends on a random data partition, so it is the only row reported as mean ± standard deviation over 5 random seeds. Best result per query type in bold.

Variant	Caption Only	Caption + Image	Image Only	Mixed (All)	Δ Mixed (pp)
Text only (α=0)	**77.00**	28.50	4.50	36.67	–
Visual only (α=1)	7.50	12.00	**11.50**	10.33	–
Both, fixed α=0.3	74.00	31.50	6.50	37.33	+0.67
Both, entropy-based α	70.50	**33.50**	8.50	37.50	+0.17
Both, learned α (ours)	**77.00 ± 0.00**	29.60 ± 1.46	10.60 ± 0.80	**38.83 ± 0.64**	+1.33

**Table 5:** Computational performance per image comparison: Standard Diffusion vs. LCM-LoRA.

Method	Latency (GPU)	Latency (CPU)	Comp. Cost
Standard Diffusion	1.25 s	6.49 min	33.93 TFLOPs
LCM-LoRA	0.31 s	2.38 min	2.71 TFLOPs
Improvement	4× speedup	2.7× speedup	12.5× reduction

**Table 6:** Clinical quality evaluation (mean ± std) by a radiologist.

Method	Mean Score	Std. Deviation
Standard Diffusion	26.65	32.96
LCM-LoRA	17.99	29.08

**Table 7:** Performance summary of the automated evaluators compared to the radiologist baseline across the 200 pairs. The metrics evaluate correlation (Pearson *r*), absolute agreement (ICC), accuracy (Mean Absolute Error, MAE) and systematic error (Mean Bias). Best result per metric type in bold.

Evaluator	Pearson *r* ↑	ICC ↑	MAE ↓	Bias → 0
Gemini 2.5 Flash	**0.805**	**0.806**	**0.143**	−0.006
Pixtral 12B	0.730	0.717	0.185	−0.052
Llama 4 Scout 17B	0.679	0.674	0.197	−0.053
CLIP score (Baseline)	0.780	0.609	0.281	+0.237

## Data Availability

The original ROCO (Radiology Objects in Context) dataset analyzed in this study is publicly available [20]. The interactive multimodal platform is publicly deployed and accessible [79].
